# Research and Remedies

**Published:** 1915-07-17

**Authors:** 


### RESEARCH AND REMEDIES.

### The Manner of Infection in Polio-Myelitis.

Flexner teaches that the infective agent of polio-myelitis is borne and disseminated by persons and not by insects. The virus of the disease has been ascertained both to enter and to leave the infected subject by way of the naso-pharyngeal mucous membrane. It has been detected in this situation not only in frank cases of infantile paralysis, but also in slight ones, abortive so-called, and even in healthy persons who have been in contact with active cases. Moreover, a few instances are known in which, after recovery, the virus was still detectable in the secretions of the naso-pharynx for several months. In other words, we have to do, in infantile paralysis, as in so many other acute infections, with a series of factors which afford potential and ready means for the dissemination far and near of the microbic agent of infection. In this distribution the abortive cases, many of which are very little ill, doubtless constitute the greatest menace, although the passive and chronic carriers of the microbes, as the healthy or recovered but contaminated persons are called, who still continue to discharge infectious secretions from the nose and throat, play each an important and sinister part in the process.—*Johns Hopkins Hospital Bulletin.*

### Traumatic Aneurisms.

The war has made these lesions prominent, and from the *Paris Médicale* we learn that in many cases treatment by operation has been very successful. M. Auvray has operated on fifteen cases (seven arterial, seven arterio-venous, and one diffuse), and all with satisfactory results, except in the one example of the diffuse variety. M. Soubeyran reports eleven instances in which the aneurism was treated by the double ligature above and below the sac, and all did well, with the exception of one case in which gaseous gangrene supervened.

### An Obstetric Accident.

Polliot, of Besançon, relates how, after a forceps delivery, the distressed father appeared before the accoucheur with an eye wrapped up in a napkin, and demanded whence it had come. As a matter of fact the infant showed, on examination, absence of one of the eyeballs, though the accoucheur had no reason to believe that he was responsible for this result. The woman had been in the hands of a midwife prior to the advent of the practitioner, and the suggestion is that she had in her vaginal examination mistaken the orbit for the anus, and had gouged out the eye.—*Quoted by Ernest Thomson in the Ophthalmoscope.*

